# Gene Polymorphisms of the Renin-Angiotensin System and Bleeding Complications of Warfarin: Genetic-Based Machine Learning Models

**DOI:** 10.3390/ph14080824

**Published:** 2021-08-22

**Authors:** Joo-Hee Kim, Jeong Yee, Byung-Chul Chang, Hye-Sun Gwak

**Affiliations:** 1 Institute of Pharmaceutical Science and Technology, College of Pharmacy, Ajou University, 206 Worldcup-ro, Yeongtong-gu, Suwon 16499, Korea; elisekim@ajou.ac.kr; 2 Graduate School of Pharmaceutical Sciences, College of Pharmacy, Ewha Womans University, 52 Ewhayeodae-gil, Seodaemun-gu, Seoul 03760, Korea; jjjhello1@naver.com; 3 Bundang CHA Medical Center, Department of Thoracic and Cardiovascular Surgery, CHA University, 59 Yatap-ro, Bundang-gu, Seongnam 13496, Korea; bcchang@cha.ac.kr; 4 Yonsei University Medical Center, Department of Thoracic & Cardiovascular Surgery, 50-1 Yonsei-ro, Seodaemun-gu, Seoul 03722, Korea

**Keywords:** haplotype, hemorrhage, machine learning, polymorphism, renin–angiotensin system, warfarin

## Abstract

This study aimed to investigate the effects of genetic variants and haplotypes in the renin–angiotensin system (RAS) on the risk of warfarin-induced bleeding complications at therapeutic international normalized ratios (INRs). Four single nucleotide polymorphisms (SNPs) of *AGT*, two SNPs of *REN*, three SNPs of *ACE*, four SNPs of *AGTR1*, and one SNP of *AGTR2*, in addition to *VKORC1* and *CYP2C9* variants, were investigated. We utilized logistic regression and several machine learning methods for bleeding prediction. The study included 142 patients, among whom 21 experienced bleeding complications. We identified a haplotype, H2 (TCG), carrying three single nucleotide polymorphisms (SNPs) of *ACE* (rs1800764, rs4341, and rs4353), which showed a significant relation with bleeding complications. After adjusting covariates, patients with H2/H2 experienced a 0.12-fold (95% CI 0.02–0.99) higher risk of bleeding complications than the others. In addition, G allele carriers of *AGT* rs5050 and A allele carriers of *AGTR1* rs2640543 had 5.0- (95% CI 1.8–14.1) and 3.2-fold (95% CI 1.1–8.9) increased risk of bleeding complications compared with the TT genotype and GG genotype carriers, respectively. The AUROC values (mean, 95% CI) across 10 random iterations using five-fold cross-validated multivariate logistic regression, elastic net, random forest, support vector machine (SVM)–linear kernel, and SVM–radial kernel models were 0.732 (0.694–0.771), 0.741 (0.612–0.870), 0.723 (0.589–0.857), 0.673 (0.517–0.828), and 0.680 (0.528–0.832), respectively. The highest quartile group (≥75th percentile) of weighted risk score had approximately 12.0 times (95% CI 3.1–46.7) increased risk of bleeding, compared to the 25–75th percentile group, respectively. This study demonstrated that RAS-related polymorphisms, including the H2 haplotype of the *ACE* gene, could affect bleeding complications during warfarin treatment for patients with mechanical heart valves. Our results could be used to develop individually tailored intervention strategies to prevent warfarin-induced bleeding.

## 1. Introduction

Warfarin has been one of the most widely used oral anticoagulants since its approval [1]. Although direct oral anticoagulants have become popular for patients who need anticoagulation therapy, warfarin remains the first-line anticoagulant for patients with heart valve prostheses [2]. Nevertheless, warfarin has several limitations, including a narrow therapeutic range and wide inter- and intra-individual variabilities [1].

Bleeding is the most serious complication of warfarin treatment [3]. Although close monitoring based on the international normalized ratio (INR) is known to be effective for evaluating the efficacy and safety of warfarin, it has been reported that patients may still experience bleeding complications within therapeutic INRs, and even at sub-therapeutic INRs [4,5]. Age, hypertension, and concomitant aspirin use—in addition to high INR—are known as patient-related risk factors for complications [3]; however, it needs to be explained further, and genetic factors can be an answer. According to Pourgholi et al., *CYP2C9* and *NQO1* variants may affect bleeding complications [6]. Our previous research also identified several gene polymorphisms (e.g., *APOB* and *GATA4*), which can affect warfarin bleedings [7,8]. However, compared to pharmacogenomic studies for warfarin dose [9], previous studies have rarely assessed the genetic effects on bleeding complications during anticoagulation therapy.

The renin–angiotensin system (RAS) consists of four major components: renin (REN), angiotensinogen (AGT), angiotensin I-converting enzyme (ACE), and angiotensin receptors (AGTR1 and AGTR2). The RAS is known to play an important role in the regulation of electrolyte balance, vasoconstriction, vascular remodeling, and fibrinolysis [10,11]. Previous studies have demonstrated clinically significant associations between RAS polymorphisms and several cardiovascular diseases (e.g., hypertension, coronary heart disease, and stroke) [12]. According to a recent meta-analysis, it has been demonstrated that *ACE* I/D polymorphism is associated with intracranial hemorrhage [13]. According to Shiotani et al., *AGT* A-20C was associated with gastrointestinal bleeding [14]. As the RAS is highly involved in cardiovascular functions, including fibrinolysis, RAS polymorphisms may affect bleeding complications.

Machine learning is a subdomain of artificial intelligence, which makes machines mimic human intelligence [15]. With powerful and fast development, machine learning has been applied to several fields, including medicine [16]. In terms of cardiology, machine learning can help clinicians by interpreting high-dimensional data (e.g., biomedical and clinical big data, multi-omics data, and images) [17] and predicting outcomes (e.g., coronary artery disease and heart failure) [18]. Several studies also have developed machine learning algorithms to predict warfarin dose [19,20,21]. However, previous studies have rarely investigated bleeding complications by applying machine learning algorithms.

Therefore, we aimed to investigate the effects of genetic variants and haplotypes of RAS-related genes on bleeding complications among mechanical heart valve patients maintaining therapeutic INRs, and we used supervised machine learning to build predictive models for bleeding occurrence.

## 2. Results

From among the 229 patients enrolled, 142 were included in the analysis. Of the 87 who were excluded, 28 did not reach stable INR, 4 had bleeding complications at supra-therapeutic INRs, and 55 reported minimal bleeding complications not verified by health professionals. Among the 55 patients who were excluded from this study because of the lack of professional verification, 8 had bleeding complications before achieving a stable INR, and 32, 6, and 9 patients had bleeding complications at therapeutic, supra-, and sub-therapeutic INRs, respectively.

As shown in Table 1, the median age and male proportion of included patients was 60 y and 36.6%, respectively. The mean follow-up period was 14.3 ± 6.4 y. Among patients included in the analysis, 21 patients reported bleedings while maintaining therapeutic INRs (11 with minor and 10 with minimal bleeding complications). Atrial fibrillation was the only significant factor for bleeding complications among demographic characteristics (*p* = 0.045).

As shown in Table 2, statistically significant associations between genotypes and bleeding complications were found for rs5050 of *AGT*, rs4341 and rs4353 of *ACE*, and rs2640543 of *AGTR1*. In the case of rs5050, 13 out of 45 patients (28.9%) with the G allele had bleeding complications, whereas 8 out of 97 patients (8.2%) with the TT genotype had bleeding complications (*p* = 0.001). For rs4341 and rs4353, patients with the wild allele were at a higher risk of bleeding, compared with those who had the variant homozygotes (19.1% vs. 6.3%, *p* = 0.041; 19.2% vs. 2.6%, *p* = 0.014, respectively). C allele carriers of rs1800764 experienced more bleeding complications than those with the TT genotype with marginal significance (18.8% vs. 6.5%, *p* = 0.055). For rs2640543, patients with the A allele had higher bleeding risks, compared with those with the GG genotype (23.4% vs. 10.5%, *p* = 0.042).

Since all of the ACE polymorphisms analyzed in the study were in a moderate linkage disequilibrium (LD) (r^2^ range: 0.63–0.79; D’ range: 0.82–0.98; Figure S1), and each SNP showed similar association with bleeding complications, we constructed haplotypes of the *ACE* gene. Five haplotypes were detected at a frequency more than 1%: H1 (CGA, 41.8%), H2 (TCG, 47.0%), H3 (TCA, 4.7%), H4 (CCG, 4.1%), and H5 (TGA, 1.4%). The most frequent haplotype was H2, containing variant alleles at every locus, and patients with H2/H2 experienced fewer bleeding complications than the others (3.0% vs. 18.3%, *p* = 0.028).

**Figure 1:**
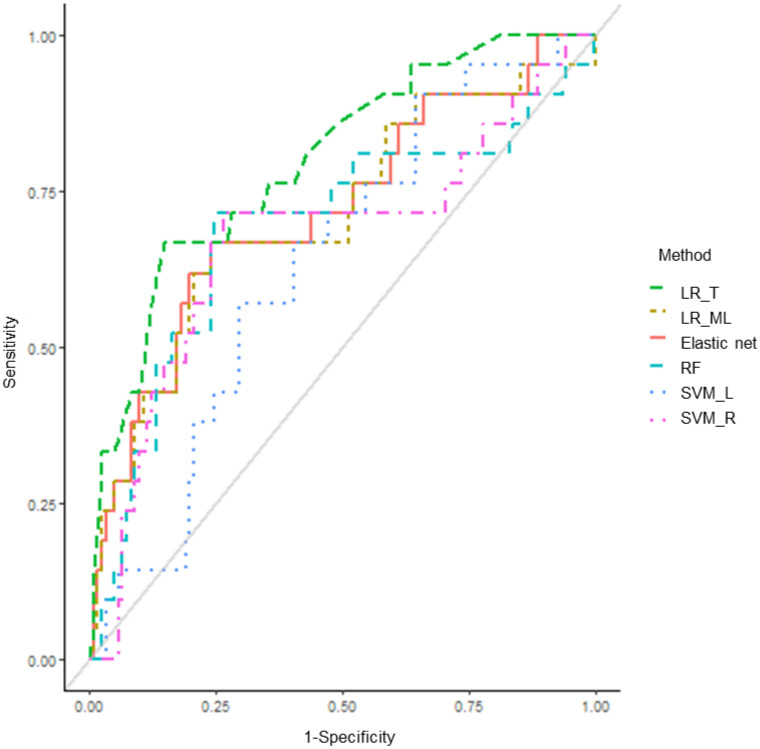
Area under the receiver operating characteristic curve (AUROC) for bleeding complications at therapeutic INRs. LR_T: traditional logistic regression; LR_ML: machine learning-based logistic regression; RF: random forest; SVM_L: support vector machine–linear kernel; SVM_R: support vector machine–radial kernel.

After adjusting for related covariates, patients with H2/H2 were revealed to have an approximately 0.12-fold higher risk of bleeding complications than the others (Table 3). Patients with G allele of rs5050 and A allele of rs2640543 had 5.0- and 3.2-fold higher rates of bleeding complications at therapeutic INRs, compared with those with the TT and GG genotypes, respectively. In constructed models, the attributable risks related to H2, rs5050 and rs2640543 were 88.0%, 80.2%, and 68.5%, respectively. The area under the receiver-operating curve (AUROC) value (mean, 95% confidence interval (CI)) was 0.771 (0.656–0.886) (Figure 1) and the Hosmer–Lemeshow test showed that the fitness of model was satisfactory (χ^2^=3.162, 5 degrees of freedom, *p* = 0.675). The AUROC values (mean, 95% CI) across 10 random iterations using five-fold cross-validated multivariable logistic regression, elastic net, random forest (RF), support vector machine (SVM)–linear kernel, and SVM–radial kernel models were 0.732 (0.694–0.771), 0.741 (0.612–0.870), 0.723 (0.589–0.857), 0.673 (0.517–0.828), and 0.680 (0.528–0.832), respectively.

We calculated the number needed to genotype (NNG) for preventing one patient with a high-risk allele or haplotype from experiencing a higher incidence of bleeding complications using each model, and the values were 8, 14, and 19 for H2, rs5050, and rs2640543, respectively. In weighted risk score (WRS) analysis, patients with bleeding complications showed a significantly higher WRS than those without (3.6 ± 1.2 vs. 2.3 ± 1.3, *p* < 0.001). As shown in Table 4, the incidence of bleeding complication displayed increasing patterns, according to the quartile of WRS. The highest quartile group (≥75th percentile) of WRS had 12.0 (95% CI 3.1–46.6, *p* < 0.001) times increased risk of bleeding, compared to the 25–75th percentile group.

## 3. Discussion

The main finding of the present study suggested that the *ACE* haplotype, H2 (TCG), carrying three SNPs (rs1800764, rs4341, and rs4353) in addition to rs5050 of *AGT* and rs2640543 of *AGTR1* were associated with bleeding complications at therapeutic INRs among patients who received warfarin therapy after mechanical heart valve replacement surgery. Patients with H2/H2 experienced a 0.12-fold higher risk of bleeding complications than the others. G allele carriers of rs5050 and A allele carriers of rs2640543 were 5.0 and 3.2 times more likely to experience bleeding complications compared with TT genotype and GG genotype carriers, respectively. In the five-fold cross-validated multivariable logistic regression, elastic net, RF, and SVM models, the mean AUROC values ranged between 0.67 and 0.74.

The RAS has essential roles in the cardiovascular system. Among its many functions, the RAS—particularly angiotensin II—is involved in vascular pathophysiology, including vascular cells growth/apoptosis, vascular smooth muscle cell differentiation/proliferation, and extracellular matrix remodeling [11]. The RAS is also known to be involved in the balance of coagulation and fibrinolysis by two processes: (1) tissue plasminogen activator (t-PA) production by bradykinin, which is metabolized by ACE, and (2) plasminogen activator inhibitor-1 (PAI-1) induction by angiotensin II [10].

The *ACE* gene, located on human chromosome 17, consists of 26 exons and 25 introns and is considered highly polymorphic. Among genetic polymorphisms of *ACE*, I/D polymorphism has been most extensively studied in several cardiovascular diseases [22,23]. Rs4341, an intronic variant of *ACE*, is known to be in perfect LD with *ACE* I/D polymorphism in Caucasian and Asian populations, and this is commonly used as an alternative method of determining *ACE* I/D polymorphism; GG genotype carriers of rs4341 are considered to be DD genotype carriers of the *ACE* I/D polymorphism [24]. In a study with healthy subjects [25], *ACE* I/D polymorphism was related to serum *ACE* concentrations, accounting for 47% of the total variance of serum ACE concentrations. A recent meta-analysis including 39 case–control studies showed that *ACE* I/D polymorphism was significantly associated with intracranial hemorrhage, especially among Asians [13]. Although some studies have described the DD genotype as a potent thrombophilic factor [26,27], several studies have reported that D allele carriers of *ACE* have increased risk of hemorrhagic stroke [28] and blood loss after hip surgery [29], which is also consistent with our results.

Rs1800764 and rs4353, included in the associated haplotype with bleeding complications in our study, were located in the upstream region and intron 19 of *ACE*, respectively. Chung et al. showed that rs1800764 had a significant relation with young-onset hypertension and rs4353 was significantly associated with ACE activity [30]. Furthermore, the haplotype containing the A allele of rs4353 was reportedly related to increased serum concentrations of ACE and increased hypertension risk [31]. eQTL analysis performed on GTEx also supported our results [32]; both rs1800764 and rs4353 were recorded as significant expression quantitative trait loci with *ACE* transcript (*p* = 3.0 × 10^−21^ and 9.8 × 10^−17^, respectively), showing higher expression with wild-type alleles in fibroblast tissues. In consideration of the above findings, our results might be explained by the increased serum concentrations of ACE.

Angiotensinogen, encoded by AGT, is the only precursor of the RAS and is sequentially cleaved by renin and ACE [33]. Gould et al. showed that plasma AGT concentrations were closely related to the K_m_ of renin, indicating that plasma AGT concentrations might affect the angiotensin II level accordingly [34]. Located in the promoter region of the *AGT* gene, rs5050 is reported to be essential for the transcription of *AGT* [35], and the variant allele of rs5050 has been reported to increase the promoter activity of *AGT* [35,36]. Ishigami et al. also revealed that this variant was associated with high *AGT* plasma concentrations [37]. In studies with patients taking low-dose aspirin, rs5050 was reportedly associated with bleeding [14,38]. Our study also revealed that this variant was significantly associated with increased bleeding risk.

AGTR1, expressed in all different organs, is the principal receptor that mediates major actions of angiotensin II [39]. Although there have been no studies that examine the bleeding risk of rs2640543, several studies have demonstrated the functional effect of rs2640543, which is linked with cardiovascular disease [40,41]. Su et al. reported that, in a study with Chinese patients, the haplotype-containing wild allele of rs2640543 was associated with decreased systolic blood pressure reduction in response to benazepril, an ACE inhibitor [40]. eQTL results also showed that the wild allele of rs2640543 also showed higher expression in both fibroblast and aorta artery (*p* = 2.5 × 10^−12^ and *p* = 3.2 × 10^−5^, respectively) [32]. Accordingly, the increased RAS activity seems to affect the bleeding.

In our study, *VKORC1* and *CYP2C9* polymorphisms, the well-known genetic factors for warfarin dose prediction [1], were not significantly associated with bleeding complications. As our study patients had already achieved therapeutic INRs after dosing adjustments, *VKORC1* and *CYP2C9* polymorphisms were expected not to affect bleeding risk.

This study applied several machine-learning-based methodologies based on the significant factors in the univariate analyses to predict bleeding complications. The AUROC values from the five-fold cross-validated multivariable logistic regression, elastic net, and RF revealed the favorable performance of these models (higher than 0.7). The elastic net is a penalized linear regression model that combines the penalties of the lasso and ridge methods [41]. RF is an ensemble method, which increases the diversity by using a random subset of available features at each node and provides a more accurate prediction than a single decision tree [42,43,44]. In the case of SVM models, the AUROC values were around 0.67. In this study, SVMs were implemented using linear and radial basis function kernels. Linear kernel SVMs have a single tuning parameter, C, which is the cost parameter of the error term, whereas radial kernel SVMs have an additional hyperparameter, sigma, which determines the width for Gaussian distribution [44,45].

Since this study dealt with patients with INRs between 2 and 3, only minimal or minor bleeding events were observed. Although it is obvious that major hemorrhages are of importance, minor bleedings are also clinically important, because they serve as warnings for subsequent major bleedings and may increase the number of visits to clinics, resulting in additional medical costs.

To evaluate the potential clinical value of genotyping SNPs, we calculated the WRS based on our logistic regression models. The highest quartile group of WRS had 12-fold significantly higher bleeding complications than the 25–75th percentile group, implying the possibility of discriminating the high-risk group of bleeding complications in patients on stable warfarin therapy.

The limitations of our study are its retrospective study design and small sample size. In addition, we did not consider the social and clinical histories (e.g., alcohol use and bleeding/stroke history), which could affect bleeding, due to the insufficient data. However, to our knowledge, this is the first study to investigate and predict the warfarin-induced bleeding complications with RAS-related genetic variants using machine learning algorithms.

## 4. Materials and Methods

### 4.1. Study Patients and Data Collection

This is a retrospective analysis of prospectively collected blood samples. The details for study patients have already been described in previous papers [7,8]. Study patients were recruited from the previous study cohort, entitled the Ewha-Severance Treatment (EAST) Group of Warfarin. Briefly, 229 patients were included who received mechanical heart valve replacement and were treated with warfarin between January 1982 and December 2009 at the Severance Cardiovascular Hospital of Yonsei University College of Medicine. Among the EAST cohort, patients with a stable INR, which was defined as at least three consecutive INR values in the therapeutic range (2–3) at the outpatient clinic, were recruited for the study. Patients whose bleedings occurred at supra- or sub-therapeutic INRs or were not confirmed by doctors were excluded.

Patients were routinely followed up at the outpatient clinic until warfarin discontinuation, loss to follow up, death, or the end of the study, whichever came first. Blood samples were collected at the outpatient visit. By reviewing patients’ medical records between January 1982 and August 2017, the following data were collected: age, sex, weight, height, body mass index, position and type of valve prosthesis, comorbidities, co-medications, follow-up time, INR values, and bleeding occurrence. Each bleeding event was confirmed by a doctor at the hospital, and the INR was checked at the time of the event. Bleeding complications were assessed using the Platelet Inhibition and Patient Outcomes (PLATO) classification (i.e., major fetal/life-threatening, major other, minor, and minimal) [46].

The Institutional Review Board of the Yonsei University Medical Center approved this study (approval number: 4-2009-0283). This study followed the ethical standards of the Institutional Review Board of the Yonsei University Medical Center and Helsinki declaration principles. Written informed consent was taken from all patients.

### 4.2. Genotyping Methods

The SNPs of RAS-associated genes were selected by reviewing the following sources: (1) Haploreg 4.1 for minor allele frequencies and LD patterns of SNPs in Asian populations [47]; (2) the PharmGKB database for clinical annotations [48]; and (3) previous studies [30,49,50]. Finally, a total of 16 SNPs were investigated: four SNPs of *AGT* (rs7079, rs699, rs5050, and rs11122576), two SNPs of *REN* (rs2368564 and rs12750834), three SNPs of *ACE* (rs1800764, rs4341, and rs4353), four SNPs of *AGTR1* (rs275651, rs2640543, rs5186, and rs5182), and one SNP of *AGTR2* (rs1403543), along with the two well-known SNPs for warfarin stable doses (*VKORC1* rs9934438 and *CYP2C9* rs1057910).

We extracted genomic DNA from patients’ blood samples using the QIAamp DNA Blood Mini Kit (QIAGEN, Hilden, Germany). All samples were genotyped using the TaqMan SNP genotyping assay (Applied Biosystems, Foster City, CA, USA) based on a real-time PCR system or SNaPShot multiplex kits (Applied Biosystems, Foster City, CA, USA) based on a single-base primer extension assay.

### 4.3. Statistical Analysis and Machine Learning Methods

We calculated the LD (r^2^ and D’) for each SNP pair in a gene by Haploview 4.2 [51] and performed haplotype analysis using Plink [52]. To compare the patients with and without bleeding complications, the chi-square test and independent t-test were used for categorical and continuous variables, respectively. To determine independent risk factors related to bleeding complications, we performed multivariable logistic regression analysis with backward elimination using variables whose *p*-value was less than 0.05 in univariate analysis, in addition to clinical confounders (age and sex). We obtained odds ratios (ORs) and adjusted odds ratios (AORs) by logistic analyses and calculated attributable risk (%) by the formula of ((AOR − 1)/AOR) × 100. The model was tested by Hosmer–Lemeshow statistics and AUROC analysis.

To predict bleeding complications, we utilized machine learning algorithms, including five-fold cross-validated multivariable logistic regression, elastic net, RF, and a SVM. In each algorithm, we used 10 repeat iterations. To evaluate model performance, we used the AUROC with a 95% CI.

The NNG, which represents the number of patients for preventing one additional bleeding complication, was calculated by the following equations [7]:

Relative risk reduction (RRR) = (AOR − 1)/AOR;

Absolute risk reduction (ARR) = RRR × Risk_no genotyping_;

NNG = 1/ARR,

where Risk_no genotyping_ was defined as the risk of higher incidence of bleeding complications without genotyping. To assess the cumulative effect of clinical factors and multiple SNPs on bleeding complications, the WRS was created based on variables that were included in each model in this study. The point assigned to each variable was determined by the variable’s beta coefficient from the logistic regression model in the current study, and the WRS was the sum of the points for variables that patients had.

A *p*-value of <0.05 was considered statistically significant. All analyses were performed with SPSS 20.0 (IBM, Armonk, NY, USA) and R package caret.

## 5. Conclusions

This study demonstrated that RAS-related polymorphisms, including the H2 haplotype of *ACE*, rs5050 of *AGT*, and rs2640543 of *AGTR1* could affect bleeding complications during warfarin treatment for patients with mechanical heart valves. Our results could be used to develop individually tailored intervention strategies to prevent warfarin-induced bleeding.

## Figures and Tables

**Table 1:** Patient characteristics of study patients.

Characteristics.	Bleeding Complication, Number (%)		*p*
	Presence (*n* = 21)	Absence (*n* = 121)	
Sex			0.705
Male	8 (38.1)	44 (36.4)	
Female	13 (61.9)	77 (63.6)	
Age (y)			0.106
<65	11 (52.4)	85 (70.2)	
≥65	10 (47.6)	36 (29.8)	
Mean ± SD	62.0 ± 11.2	58.7 ± 10.0	0.168
Body weight (kg) Mean ± SD	58.6 ± 10.7	58.7 ± 10.4	0.989
Body mass index (kg/m^2^) Mean ± SD	22.3 ± 2.3	22.5 ± 2.8	0.756
Comorbidity			
Hypertension	6 (28.6)	33 (27.3)	0.902
Diabetes mellitus	3 (14.3)	10 (8.3)	0.377
Chronic heart failure	7 (33.3)	25 (20.7)	0.199
Atrial fibrillation	17 (81)	70 (57.9)	0.045
Myocardial infarction	2 (9.5)	2 (1.7)	0.104
Comedication			
Angiotensin-converting-enzyme inhibitor	2 (10.5)	19 (18.8)	0.383
Angiotensin II receptor blocker	4 (21.1)	19 (18.8)	0.820
Antiplatelet drugs ^a^	0 (0)	4 (3.8)	0.398
Calcium channel blocker	4 (21.1)	19 (18.8)	0.820
Diuretics	9 (47.4)	35 (34.7)	0.291
Statins	0 (0)	4 (4.0)	0.378
INR increasing drugs ^b^	0 (0)	1 (1.0)	1.000
INR decreasing drugs ^c^	1 (5.3)	0 (0)	1.000
Valve position			0.740
Aortic	6 (28.6)	28 (23.1)	
Mitral	9 (42.9)	66 (54.5)	
Double ^d^	5 (23.8)	20 (16.5)	
Tricuspid ^e^	1 (4.8)	7 (5.8)	
Valve type			0.418
St. Jude Medical	7 (38.9)	39 (34.2)	
CarboMedics	6 (33.3)	32 (28.1)	
ATS	2 (11.1)	15 (13.2)	
MIRA	1 (5.6)	9 (7.9)	
Duromedics	2 (11.1)	6 (5.3)	
OnX	0 (0)	4 (3.5)	
Others ^f^	0 (0)	9 (7.9)	
INR Mean ± SD	2.41 ± 0.07	2.45 ± 0.10	0.143
Follow-up time (y) Mean ± SD	14.27 ± 6.20	14.48 ± 7.59	0.886

INR: international normalized ratio; ^a^ aspirin, clopidogrel, and cilostazol, ^b^ amiodarone, fluconazole, and NSAID, ^c^ carbamazepine, phenytoin, and rifampin, ^d^ aortic plus mitral valve, ^e^ tricuspid valve with or without other valves, ^f^ including Sorin, Bjork Shiley, D-ring, and prostheses using two or more different valve types.

**Table 2:** Genetic factors associated with bleeding complications at therapeutic INR.

Gene Polymorphism	Allele Change	Minor Allele Frequency	Grouped Genotypes	Bleeding Complication, Number (%)		*p*
				Presence (*n* = 21)	Absence (*n* = 121)	
*VKORC1*	C>T	0.113	CC, CT	3 (14.3)	27 (22.3)	0.405
rs9934438			TT	18 (85.7)	94 (77.7)	
*CYP2C9*	A>C	0.043	AA	18 (85.7)	111 (92.5)	0.304
rs1057910			AC	3 (14.3)	9 (7.5)	
*AGT*	G>T	0.128	GG	19 (90.5)	87 (72.5)	0.079
rs7079			GT, TT	2 (9.5)	33 (27.5)	
*AGT*	A>G	0.180	AA, AG	5 (23.8)	42 (34.7)	0.327
rs699			GG	16 (76.2)	79 (65.3)	
*AGT*	T>C	0.401	TT, TC	19 (90.5)	99 (81.8)	0.529
rs11122576			CC	2 (9.5)	22 (18.2)	
*AGT*	T>G	0.165	TT	8 (38.1)	89 (73.6)	0.001
rs5050			TG, GG	13 (61.9)	32 (26.4)	
*REN*	C>T	0.225	CC	14 (66.7)	71 (58.7)	0.491
rs2368564			CT, TT	7 (33.3)	50 (41.3)	
*REN*	G>A	0.373	GG	7 (33.3)	52 (43.0)	0.408
rs12750834			GA, AA	14 (66.7)	69 (57.0)	
*ACE*	C>T	0.465	CC, CT	18 (85.7)	78 (64.5)	0.055
rs1800764			TT	3 (14.3)	43 (35.5)	
*ACE*	G>C	0.437	GG, GC	18 (85.7)	76 (62.8)	0.041
rs4341			CC	3 (14.3)	45 (37.2)	
*ACE*	A>G	0.486	AA, AG	20 (95.2)	84 (69.4)	0.014
rs4353			GG	1 (4.8)	37 (30.6)	
*AGTR1*	T>A	0.094	TT	17 (85.0)	97 (81.5)	1.000
rs275651			TA, AA	3 (15.0)	22 (18.5)	
*AGTR1*	A>G	0.190	AA, AG	11 (52.4)	36 (29.8)	0.042
rs2640543			GG	10 (47.6)	85 (70.2)	
*AGTR1*	C>T	0.254	CC	3 (14.3)	6 (5.0)	0.130
rs5182			CT, TT	18 (85.7)	115 (95.0)	
*AGTR1*	A>C	0.060	AA	18 (85.7)	107 (88.4)	0.718
rs5186			AC	3 (14.3)	14 (11.6)	
*AGTR2*	G>A	0.310	GG	3 (14.3)	20 (16.5)	1.000
rs1403543			GA, AA	18 (85.7)	101 (83.5)	

**Table 3:** Multivariable analysis to identify predictors for bleeding complications at therapeutic INR.

Variables	Unadjusted OR (95% CI)	Adjusted OR (95% CI)	Attributable Risk (%)
Male	1.08 (0.41–2.80)		
Age ≥ 65 y	2.15 (0.84–5.50)		
Atrial fibrillation	3.10 (0.98–9.75)		
*ACE* H2/H2	0.14 (0.02–1.08)	0.12 (0.02–0.99) *	88.0 ^a^
*AGT* rs5050 G allele	4.52 (1.72–11.91)	5.04 (1.80–14.11) **	80.2
*AGTR1* rs2640543 A allele	2.60 (1.01–6.65)	3.17 (1.13–8.89) *	68.5

H2: T-C-G (rs1800764-rs4341-rs4353); ^a^ Attributable risk for not having *ACE* H2/H2; * *p* < 0.05, ** *p* < 0.01.

**Table 4:** Weighted risk score analysis of patients with bleeding complications.

Weighted Risk Score Percentile	Bleeding Complications		Odds Ratio (95% CI)
	Presence	Absence	
≤25th percentile	1 (3.4)	28 (96.6)	0.25 (0.03–1.95)
25–75th percentile	13 (12.7)	89 (87.3)	1 (reference)
≥75th percentile	7 (63.6)	4 (36.4)	11.98 (3.08–46.65) *

The 25th and 75th percentile of weighted risk score were 2 and 4; * *p* < 0.001.

## Data Availability

The datasets used and/or analyzed during the current study are available in the Mendeley Data repository, http://dx.doi.org/10.17632/s5cngbxmy7.1 (accessed on 20 August 2021).

## Supplementary Materials

The following are available online at https://www.mdpi.com/article/10.3390/ph14080824/s1, Figure S1. Linkage disequilibrium patterns and relative position of SNPs among study patients. Present SNPs were in *AGT* ((a) and (e)), *REN* ((b) and (f)), *ACE* ((c) and (g)) and *AGTR1* ((d) and (h)), respectively. Values in squares are the pairwise calculation of D’ ((a)–(d)) or r^2^ ((e)–(h)). Empty squares indicate D’ = 1.0 or r^2^ = 1.0.

## Abbreviations

ACE	angiotensin I-converting enzyme
AGT	angiotensinogen
AGTR	angiotensin receptor
AORs	adjusted odds ratios
AUROC	area under the receiver-operating curve
INRs	international normalized ratios
LD	linkage disequilibrium
NNG	number needed to genotype
ORs	odds ratios
PAI-1	plasminogen activator inhibitor-1
PLATO	Platelet Inhibition and Patient Outcomes
RAS	renin–angiotensin system
REN	renin
RF	random forest
SVM	support vector machine
SNPs	single nucleotide polymorphisms
WRS	weighted risk score
