# Supplementary material for: Gene Polymorphisms of the Renin-Angiotensin System and Bleeding Complications of Warfarin: Genetic-Based Machine Learning Models
Source: Pharmaceuticals (Basel). 2021 Aug 22;14(8):824. doi: 10.3390/ph14080824 (PMC8400908; doi:10.3390/ph14080824)
Supplement: Supplementary file 1 [file pharmaceuticals-14-00824-s001.zip › pharmaceuticals-1310359-supplementary.pdf]

Genetic variants and haplotypes in the renin–angiotensin system could affect bleeding complications among Korean mechanical heart valve patients on warfarin

Joo Hee Kim, PharmD, PhD[1,2*], Jeong Yee, PhD[1*], Byung Chul Chang, MD, PhD[3,4], Jee Eun Chung, PhD[5], Kyung Eun Lee[6], Hye Sun Gwak, PharmD, PhD[1].

[1]College of Pharmacy and Graduate School of Pharmaceutical Sciences, Ewha Womans University, 52 Ewhayeodae-gil, Seodaemun-gu, Seoul 03760, Korea

[2]College of Pharmacy & Institute of Pharmaceutical Science and Technology, Ajou University, 206 Worldcup-ro, Yeongtong-gu, Suwon 16499, Korea

[3]Department of Thoracic and Cardiovascular Surgery, Bundang CHA Medical Center, CHA University, 59 Yatap-ro, Bundang-gu, Seongnam 13496, Korea

[4]Department of Thoracic & Cardiovascular Surgery, Yonsei University Medical Center, 50-1 Yonsei-ro, Seodaemun-gu, Seoul 03722, Korea

[5]College of Pharmacy, Hanyang University, 55 Hanyangdeahak-ro, Sangnok-gu, Ansan 15588, Korea

[6]College of Pharmacy, Chungbuk National University, 660-1, Yeonje-ri, Osong-eup, Heungdeok-gu, Cheongju 28160, Korea

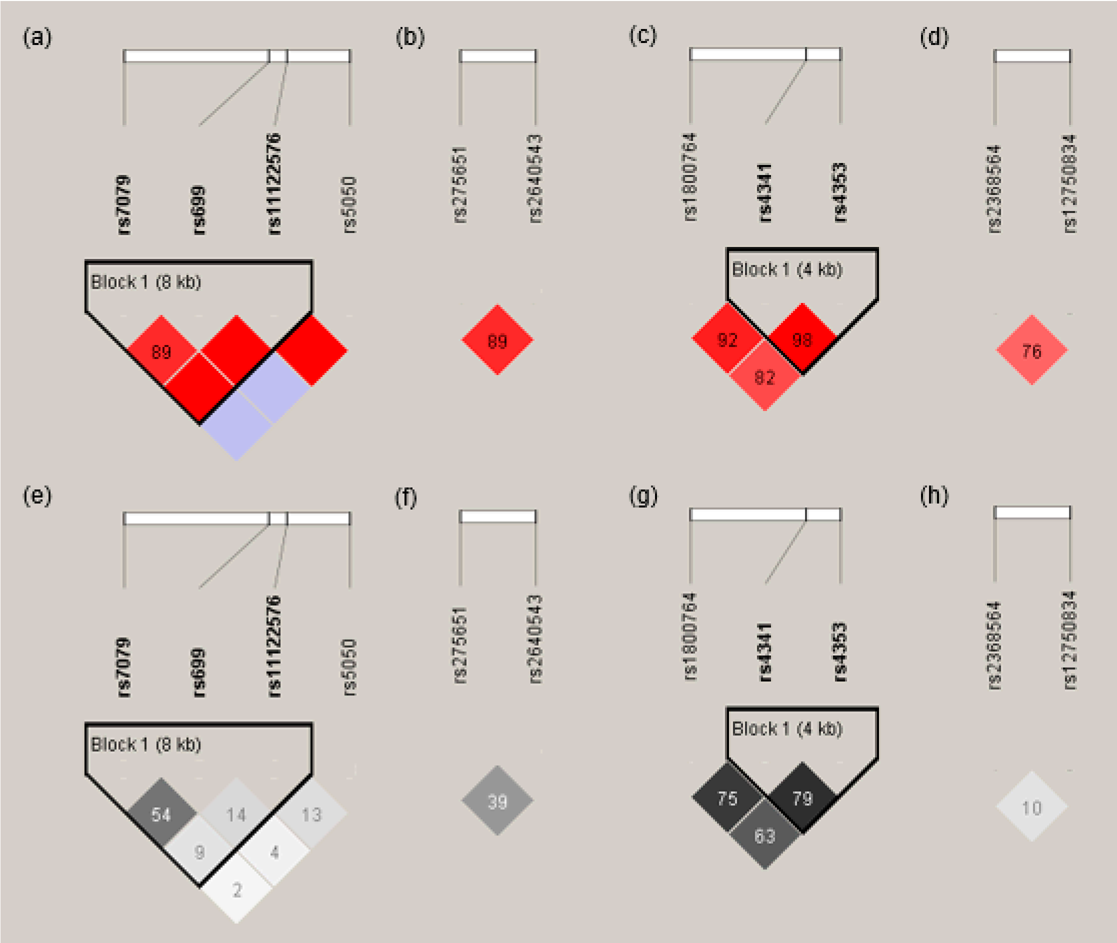

Supplementary Figure S1. Linkage disequilibrium patterns and relative position of SNPs among study patients. Present SNPs were in *AGT* ((a) and (e)), *REN* ((b) and (f)), *ACE* ((c) and (g)) and *AGTR1* ((d) and (h)), respectively. Values in squares are the pairwise calculation of D' ((a)-(d)) or r^2 ((e)-(h)). Empty squares indicate D'=1.0 or r^2=1.0.

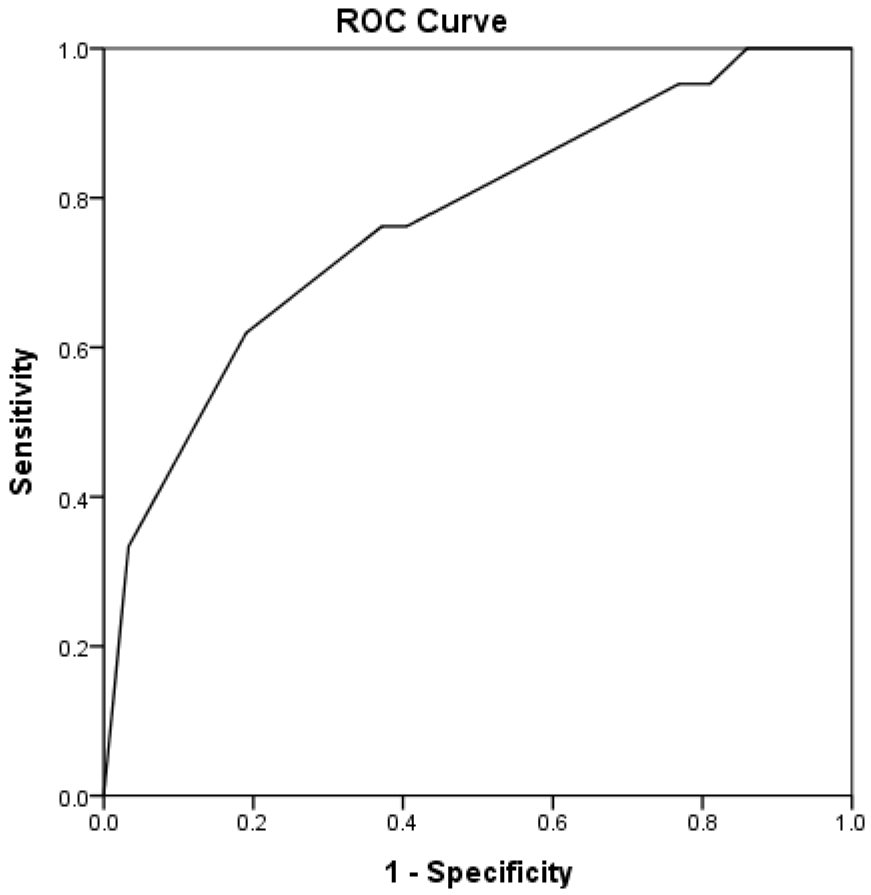

Supplementary Figure S2. Area under the receiver operating characteristic curve (AUROC) for bleeding complications at therapeutic INRs. The AUROC value was 0.771 (95% CI 0.656-0.886, $p < 0.001$).
